# Cost-effectiveness of leadless versus transvenous single-chamber ventricular pacing: a propensity-weighted real-world study in France

**DOI:** 10.1080/07853890.2026.2652657

**Published:** 2026-04-06

**Authors:** François Avry, Philippe Tessier, Hugo Fourniguet, Nicolas Arlicot, Jean Capsec, Stéphanie Bénaïn, Youssouf Compaoré, Alexandre Bodin

**Affiliations:** a Hospital Pharmacy, University Hospital Centre of Tours, Chambray les Tours, France; b SPHERE, Nantes University, INSERM, Methods in Patients-Centered Outcomes and Health Research, Nantes, France; c iBraiN, University Hospital Centre of Tours, INSERM, Tours, France; d University of Tours, INSERM, Tours, France; e Medical Information Department, Epidemiology & Health Economics, University Hospital Centre of Tours, France; f Health Technology Assessment and Healthcare Product Development Unit (SEEDePS), Nantes University Hospital, Nantes, France; g Cardiology Department, University Hospital Centre of Tours, Chambray les Tours, France

**Keywords:** Cost effectiveness, leadless pacemaker, weighted population, sensibility analysis

## Abstract

**Background:**

Leadless pacemakers (LP) have emerged as an alternative to conventional transvenous single-lead ventricular pacemakers (SLP), with expanding indications beyond initially selected patients. This study evaluated the clinical and economic outcomes of LP compared with SLP in eligible patients from a real-world French hospital cohort.

**Methods:**

We conducted a retrospective, propensity-weighted cost-effectiveness analysis from a hospital-based perspective, including all patients who underwent first LP or SLP implantation between 2016 and 2020 at our center. Patients with contraindications or high risk of device failure or infection were excluded. Four-year costs and life-years were used to estimate the incremental cost-effectiveness ratio (ICER). Cost-effectiveness acceptability curves (CEACs) were derived from the bootstrapped distribution of net monetary benefits. Sensitivity analyses explored cost drivers and robustness.

**Results:**

A total of 352 patients were included (104 LP, 248 SLP). After weighting, LP was associated with shorter procedure duration (52 vs 80 min; *p* < 0.001) and hospital stay (3.6 vs 4.3 days; *p* = 0.007). Major clinical events occurred in 25.2% (LP) vs 33.8% (SLP; *p* = 0.17). Although implantation costs were higher for LP (€11,344 vs €9,326; *p* < 0.001), total 4-year costs were comparable (€12,925 vs €11,713; *p* = 0.13). The survival difference was 0.06 years (*p* = 0.67), yielding an ICER of €20,387 per life-year gained. Bootstrap analysis indicated a 70% probability of cost-effectiveness at a €35,000 per life-year threshold.

**Conclusions:**

In both device eligible patients, leadless pacemakers showed favorable procedural and clinical outcomes with comparable long-term costs, supporting their cost-effective use in real-world French hospital practice.

## KEY LEARNING POINTS

**What is already known**

- Leadless pacemakers (LPs) were developed to overcome complications associated with conventional single-lead transvenous pacemakers (SLPs), such as pocket infection, lead dislodgement, and pneumothorax.
- LPs have demonstrated lower complication and reintervention rates, as well as improved long-term survival in high-risk populations (e.g. dialysis or post–lead extraction patients).
- Despite these advantages, their higher initial cost has limited widespread adoption, particularly among patients eligible for both pacing systems.

**What this study adds**

- This real-world, weighted cost-effectiveness analysis compares LP and SLP implantation over a 4-year follow-up in a population eligible for both devices from a hospital perspective.
- The reduction in complications, operative time, and length of stay offsets approximately 65% of the initial additional cost of LPs in France.
- Although survival benefit remains uncertain at 4 years due to wide confidence intervals, the incremental cost-effectiveness ratio (ICER) appears reasonable, with a 70% probability of cost-effectiveness. The overall value of LPs is strongly influenced by device price and by the time horizon considered.

## GRAPHICAL ABSTRACT

**Figure:**
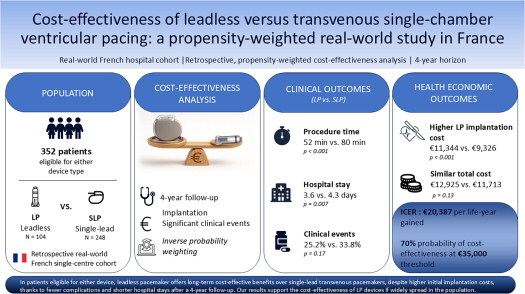

## Introduction

With the aging of the population and the growing prevalence of cardiac conduction disorders, the demand for cardiac pacing continues to rise. Conventional single lead transvenous ventricular pacemakers (SLPs), although widely used, are associated with notable short- and long-term complications in approximately 6 to 12% of patients [1–3], including pocket infection, pneumothorax, hematoma, lead dislodgment, and heart failure [1,2,4,5]. These complications are not only clinically significant but also financially burdensome, with corresponding management costs for the French national social security system over three years reaching up to €6,674 ± €3,867 per patient [6] – and over €23,000 in cases of device-related infection [7].

Leadless pacemakers (LPs) were introduced as a response to these limitations. One of the first LP, the Micra VR^®^ received FDA approval in 2016, offering right ventricular pacing *via* a catheter-based femoral approach. Early data suggested a reduction in complication rates compared to historical SLP cohorts [8–10], with lower reintervention rate at two years [11]. Importantly, LPs have been associated with improved long-term survival outcomes [12,13], particularly in specific high-risk populations such as patients undergoing hemodialysis (HR = 0.68, 95% CI [0.47–0.99], *p* < 0.05) [14], and those with a history of lead extraction (RR = 0.30, 95% CI [0.14–0.68], *p* < 0.01) [15].

However, most of these studies focus on high-risk patients – those with previous infections, venous obstruction, or chronic hemodialysis [16–21] – limiting the generalizability of findings. As a result of this restricted patient selection, it remains unclear whether LPs provide a significant clinical benefit and may prove cost-effective in patients who are eligible for both pacing systems.

Given the nearly two-fold cost of LPs compared to SLPs in France, assessing their cost-effectiveness in broader, more typical populations is critical. This is especially relevant as the global single-chamber pacemaker market is expected to grow from $890 million in 2018 to over $1.7 billion by 2027 in Europe [22]. In this context of expanding indications and increasing financial pressure on healthcare systems, providing real-world economic information is essential to guide evidence-based device selection and ensure optimal resource allocation.

In this study, we aim to evaluate the retrospective cost-effectiveness of SLP compared to LP implantation in a homogeneous patient cohort.

## Methods

### Study design

This cost-effectiveness analysis was conducted retrospectively using data on patients’ hospital resource utilization and survival, extracted from the hospitalization database (*‘Programme de Médicalisation des Systèmes d’Information’, PMSI*) of the University Hospital Centre of Tours (France). It followed the CHEERS reporting guidelines and adopted a hospital system perspective, as recommended by French National guidelines on economic evaluation in health care [23]. A 4-year time horizon was deemed appropriate to capture a substantial proportion of clinical events and associated costs.

This retrospective study was approved by the local Institutional Review Board of Tours University Hospital Centre under the French MR-004 regulatory framework. Data collection and management were conducted in accordance with CNIL requirements (authorization number 2023_080). In line with French legislation governing retrospective, non-interventional studies, the requirement for individual written informed consent was waived. Patients were systematically informed, at the time of care and through institutional information notices displayed within hospital departments, of the potential secondary use of their anonymized clinical data for research purposes and were given the opportunity to object (opt-out procedure). No patient expressed opposition to the use of their data for this study. All procedures were performed in compliance with the Declaration of Helsinki.

### Study population and settings

We included all patients who underwent primary implantation of either a SLP or an LP between October 1, 2016, and December 31, 2020, at Tours University Hospital. The first 60 LP implantations performed in 2016 were excluded, as they were considered part of the procedural learning curve (four operators). Patient data were collected from the institutional database, including demographics, comorbidities, pacing indications, and clinical events. The Charlson Comorbidity Index was calculated to assess patients’ baseline clinical status.

Patients were excluded if they required an atrial lead for AAI(R) or DDD(R) pacing, or if cardiac resynchronization therapy (CRT) was indicated. Additional exclusion criteria included restrictions for transvenous single-chamber pacemaker implantation, such as absence of supra-caval venous access, active infectious risk (history of device infection, endocarditis, or sepsis), or high complication risks (hemodialysis, implanted chemotherapy port, or prior lead failure/dislodgment). Patients with an ongoing infectious syndrome at the time of implantation (e.g. fever, elevated CRP, or confirmed viral infection) were also excluded.

We excluded all patients who were still alive at the end of the 4-year follow-up but <1 medical check-up at our center during that period.

Any patient who was not classified in the french DRG code 05C15 *‘Poses d’un stimulateur cardiaque permanent sans infarctus aigu du myocarde, ni insuffisance cardiaque congestive, ni état de choc’* (Implantation of a permanent pacemaker without acute myocardial infarction, congestive heart failure, or cardiac shock) or code 05C14 *‘Poses d’un stimulateur cardiaque permanent avec infarctus aigu du myocarde ou insuffisance cardiaque congestive ou état de choc’* (Implantation of a permanent pacemaker with acute myocardial infarction, congestive heart failure, or cardiac shock) and presenting myocardial infarction management concurrent to the primo pacemaker implantation stay were also excluded.

### Costs

Due to the use of the PMSI, only hospital costs were considered, and all costs are reported in 2022 euros (€).

A micro-costing approach was applied to estimate the cost of the index hospitalization for pacemaker implantation. Data sources included the PMSI database for length of stay, local hospital records for operating theatre occupancy times, and the Pharma^®^ traceability software (Computer Engineering, Paris, France) for implanted devices. Unit costs were provided by the Tours University Hospital Finance and Accounting Control Department, based on the hospital’s productivity register.

The average daily cost of hospitalization (ADC) was calculated by dividing the annual expenditures of the cardiology department – including staff salaries, drugs, catering, consumables, accommodation, and medical record logistics, but excluding implantable medical devices – by the total number of hospitalization days. The hourly cost of operating theatre use (HCU) was derived from the expenditures of the electrophysiology operating rooms where procedures were performed. Both direct (staff, drugs, catering, financial charges, depreciation) and indirect (logistics, technical support, administration) expenditures were taken into account, resulting in final estimates of €1,190 per day for hospitalization and €895 per hour for operating theatre use. Implanted devices were valued according to the latest public procurement prices. All device costs related to implantation failures were included.

The total cost of the index hospitalization was calculated as the sum of hospitalization costs, operating theatre costs, and device-related costs. Drug expenditures related to patient management during hospitalization were considered comparable across groups and therefore did not influence cost differentials.

Costs related to complications and clinical events were estimated using French diagnosis-related group (*DRG; Groupe Homogène de Malade, GHM*) codes extracted from the national hospital discharge database (PMSI). DRG-specific cost estimates were derived from the French National Cost Study (*Etude Nationale des Coûts, MCO*) [24]. All identified hospitalizations related to a recorded complications were included in the cost assessment, including device upgrade, venous thrombosis, stroke, pulmonary embolism, pneumothorax or hemothorax, arteriovenous fistula, pacemaker syndrome, pericardial perforation, pocket infection, sepsis, endocarditis, hematoma, major bleeding, lead dysfunction, device migration and device erosion. Hospitalizations for heart failure were also considered clinical events and were included in the cost analysis. However, heart failure admissions occurring in the context of a documented infectious event were excluded, as they were deemed unlikely to be directly attributable to device-related complications.

### Outcomes

Life-years (LYs) were used as the outcome to assess the effectiveness of each cardiac pacing system on all-cause mortality. Survival time was measured from device implantation until death or censoring at the end of follow-up. This information was cross-checked and compiled from both the hospital’s local database and the French Register of Deceased Persons, compiled by the French National Institute for Statistics and Economic Studies (INSEE) [25,26].

### Cost-effectiveness analysis

Cost-effectiveness was assessed by calculating the incremental cost-effectiveness ratio (ICER), defined as the difference in IPW-adjusted mean total costs per arm divided by the corresponding IPW-adjusted mean difference in life-years (LYs) between the LP and SLP groups. Both costs and Lys were discounted at an annual rate of 2.5%.

### Statistical analysis

Baseline clinical variables, comorbidities, complications, and cost data were first analyzed in their unweighted form. Categorical variables are presented as frequencies and percentages, and continuous variables as means with standard deviations. Group comparisons were performed using Student’s t-test or Mann–Whitney U test for continuous variables, depending on distribution, and Pearson’s chi-square test or Fisher’s exact test for categorical variables, as appropriate.

To adjust for covariates between two groups, normalized inverse probability weighting (IPW) based on propensity scores was applied [27,28]. Baselines characteristics were selected through stepwise logistic regression with a significance threshold of 0.3. Balance diagnostics included graphical assessments and standardized mean differences. Multicollinearity was evaluated using variance inflation factors, and model specification was checked *via* link tests. Weighted population categorical variables were analyzed using Pearson’s chi-square test or Fisher’s exact test, as appropriate and continuous variables were analyzed with Student’s t-test ponderated. The proportional hazards assumption was assessed using Kaplan–Meier curves and scaled Schoenfeld residuals.

Statistical uncertainty around the ICER was assessed *via* a stratified non-parametric bootstrap (10 000 replications). In each replicate, propensity scores were re-estimated by stepwise logistic regression (*p* < 0.30), IPWs were truncated to [0.15, 7.00], and cost and effectiveness differences and the ICER were computed. 95% CIs were defined by the 2.5th and 97.5th percentiles of these distributions. Cost-effectiveness acceptability curve (CEAC) was generated to illustrate the probability that LP Micra VR^®^ was cost-effective, i.e. that the difference between monetized effectiveness and costs was superior to zero, across a range of willingness-to-pay (WTP) thresholds per life-year gained. For each WTP value (from €0 to €80,000 in €1,000 increments), the proportion of bootstrap replications in which Micra was cost-effective was calculated. These probabilities were plotted to produce the CEAC. For all tests performed, a p-value < 0.05 was considered statistically significant. All statistical analyses were performed using Stata version 18.5.

### Sensitivity analysis

A one-way deterministic sensitivity analysis was performed to assess the robustness of the base-case CEA. Parameters were varied one at a time while all other inputs remained at their base values. Plausible variations were chosen to reflect parameter uncertainty and realistic implementation scenarios, including changes in the discount rate, fluctuations in device procurement costs, potential efficiency gains in length of stay and implantation procedures, and possible improvements in LP effectiveness. In addition, a scenario excluding hospitalizations related to heart failure exacerbations during follow-up was tested. The baseline IPW-weighted bootstrapped dataset was recalculated using these alternative parameter values, and cost-effectiveness acceptability curves were subsequently derived.

## Results

### Population and balance

**Figure 1.**
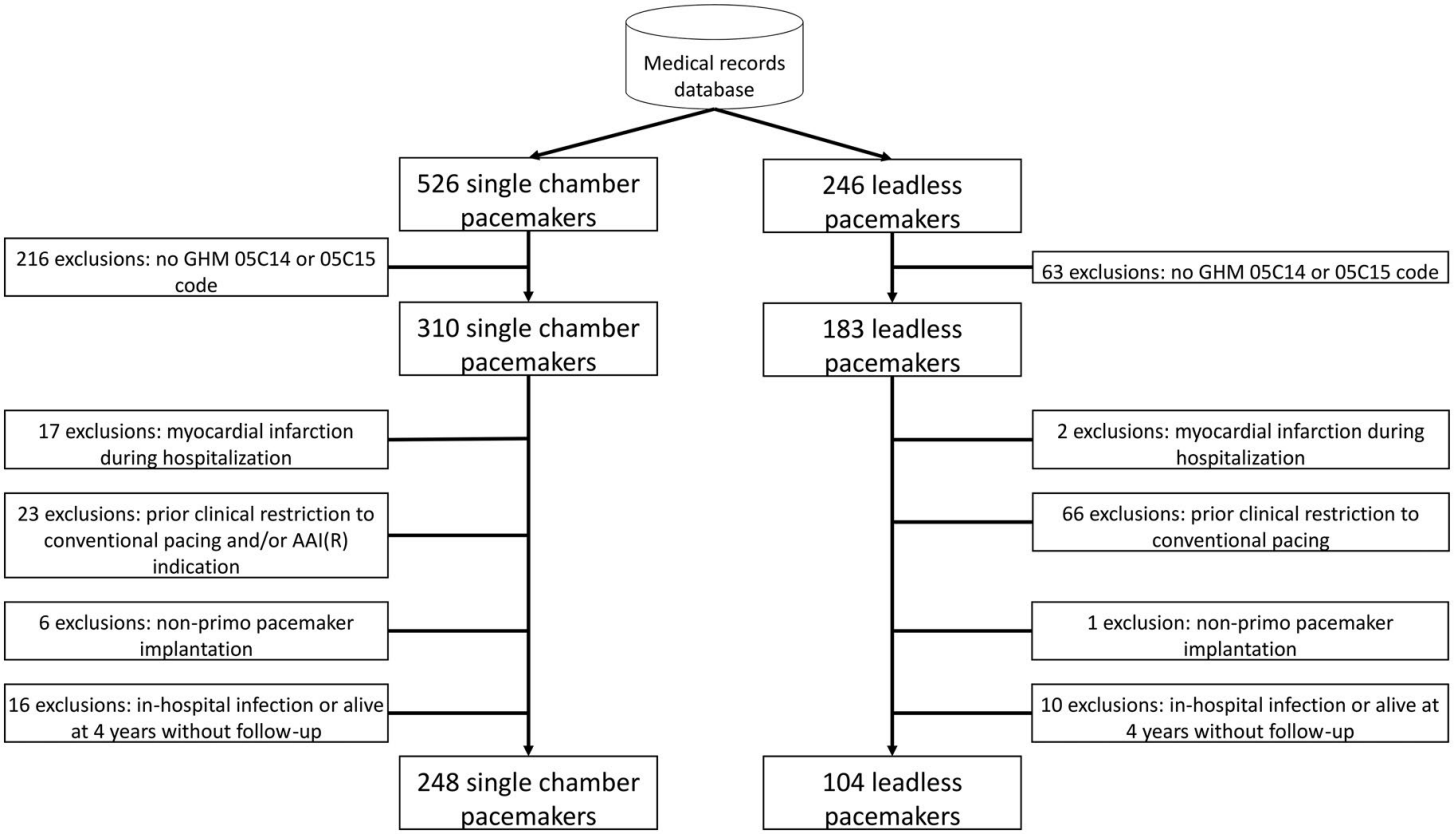
Population selection flowchart.

**Figure 2.**
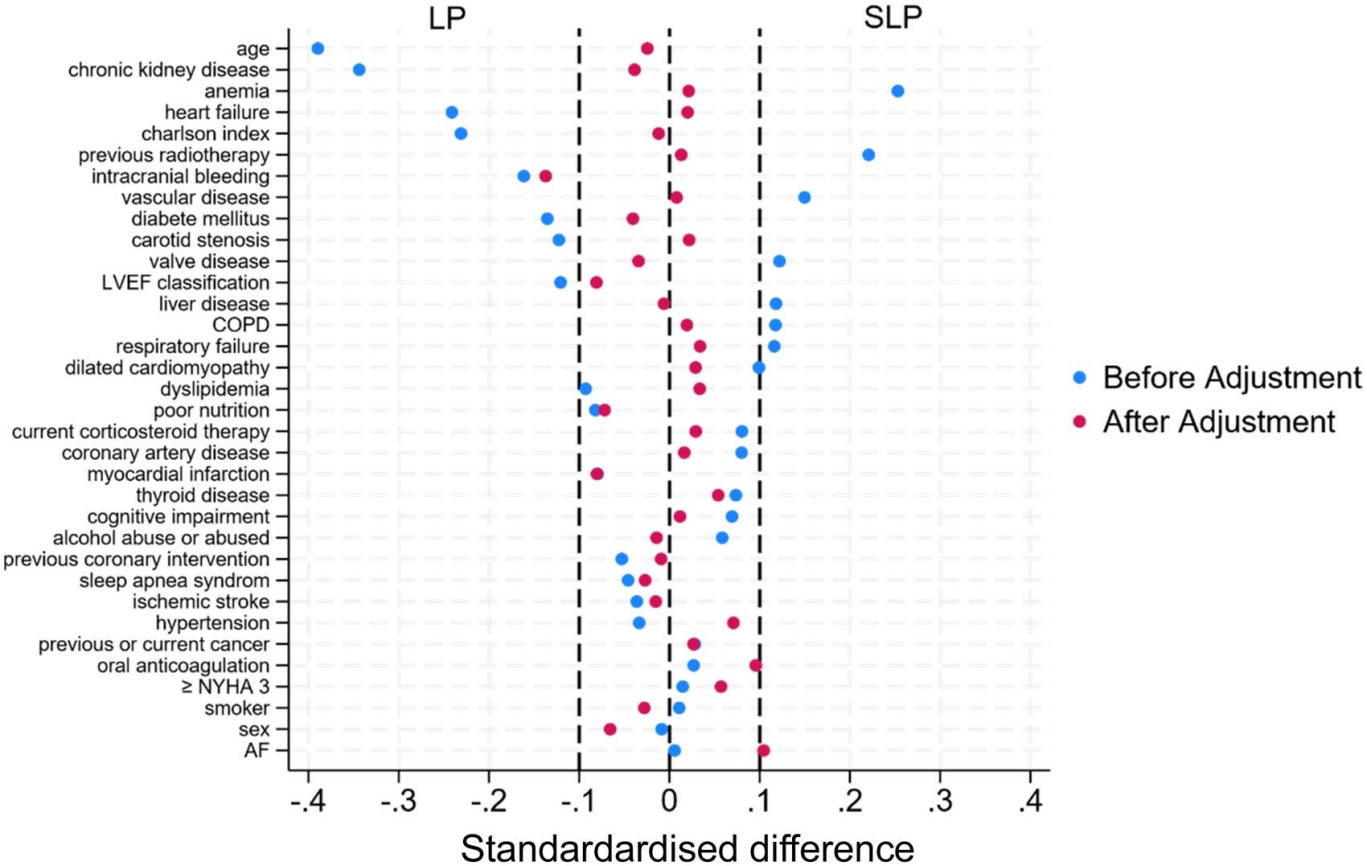
Love plot before and after IPW.

From the 772 patients extracted from the local database, 352 patients were included (Figure 1), with 104 in the LP group and 248 in the SLP group. Prior to weighting, LP patients were younger (79.0 ± 12.7 vs. 83.5 ± 10.2 years, *p* < 0.001), with fewer cases of chronic kidney disease (27.2% vs. 44.2%, *p* = 0.003) and heart failure (57.8% vs. 70.0%, *p* = 0.03), but SLP group had more frequent anemia (30.5% vs. 18.2%, *p* = 0.01) (Table 1). After applying propensity score weighting, baseline characteristics were well balanced (standardized differences mostly ≤ 0.1; Table 2, Figure 2).

**Table 1.** Baseline clinical characteristics before weighting.

Variable	LPM (*n* = 104)	SCP (*n* = 248)	*p*
**Baseline Characteristics**			
Age (Years)	79.0 (12.7)	83.5 (10.2)	<0.001
Sex, Men	57 (54.8%)	137 (55.2)	0.94
Hypertension	92 (88.5)	222 (89.5)	0.77
Diabetes Mellitus	19 (18.3)	59 (23.4)	0.26
Heart Failure	59 (57.8)	173 (70.0)	0.03
Valve Disease	29 (27.9)	56 (22.6)	0.29
Dilated Cardiomyopathy	18 (17.5)	34 (13.8)	0.38
Vascular Disease (Aneurysm, Peripheral Occlusive Disease, Vasculitis, Enterectomy)	21 (20.2)	36 (14.5)	0.19
Carotid Stenosis	8 (7.7)	28 (11.3)	0.31
Coronary Artery Disease	31 (29.8)	65 (26.2)	0.49
Previous Myocardial Infarctus	10 (9.6)	10 (9.6)	0.5
Chronic Kidney Disease	28 (27.2)	107 (44.2)	0.003
Previous Coronary Intervention	14 (13.5)	38 (15.3)	0.65
Ischemic Stroke	15 (14.4)	39 (15.7)	0.76
Intracranial Bleeding	2 (1.9)	12 (4.8)	0.25
Smoker	21 (20.2)	49 (19.8)	0.92
Dyslipidemia	48 (46.2)	126 (50.8)	0.43
Liver Disease	3 (2.9)	3 (1.2)	0.37
Alcohol Abuse	5 (4.8)	9 (3.6)	0.61
Thyroid Disease	17 (16.4)	34 (13.7)	0.52
Poor Nutrition	2 (1.9)	8 (3.2)	0.73
Previous Cancer	17 (16.4)	38 (15.3)	0.81
AF	77 (74.0)	183 (73.8)	0.96
Anemia	29 (30.5)	43 (18.2)	0.01
≥ NYHA 3	33 (31.7)	77 (31.1)	0.9
Sleep Apnea Syndrom	26 (25.2)	67 (27.0)	0.73
Respiratory Failure	11 (10.6)	18 (7.3)	0.3
COPD	8 (7.7)	12 (4.8)	0.29
Cognitive Impairment	10 (9.6)	19 (7.7)	0.54
Pacing Indication			0.28
AV Block Without Sinusal Rythm	44 (42.3)	118 (47.6)	
AV Node Ablation	30 (28.9)	63 (25.4)	
Paroxysmal AV Block In Sinusal Rythm With Low Ventricular Stimulation	20 (19.2)	34 (13.7)	
Sinus Node Dysfunction	7 (6.7)	14 (5.7)	
AV Block In Sinusal Rythm When AV Synchronisation Is Not Required	3 (2.9)	19 (7.7)	
LVEF Classification			0.52
1	89 (86.4)	196 (81.3)	
2	10 (9.7)	32 (13.3)	
≥ 3	4 (3.9)	13 (5.4)	
Previous Radiotherapy	6 (5.8)	4 (1.6)	0.07
Anticoagulation	73 (70.2)	171 (69.0)	0.82
Corticoid Therapy	5 (4.8)	8 (3.2)	0.54
Charlson Comorbidity Index	4.7 (1.8)	5.1 (1.7)	0.21
Mean Survival, Years	3.30 (1.32)	3.06 (1.33)	0.13
Length Of Stay (Days)	3.8 (2.4)	4.4 (2.4)	<0.001
Duration Of Implantation (Min)	52.3 (15.2)	79.0 (20.4)	<0.001

**Table 2.** Baseline clinical characteristics after weighting.

Variable	LPM (*n* = 104)	SCP (*n* = 248)	*p*
**Baseline Characteristics**			
Age (Years)	82.0 (10.2)	82.3 (11.6)	0.83
Sex, Men	54.5	57.8	0.62
Hypertension	91.1	88.9	0.54
Diabetes Mellitus	20.5	22.1	0.77
Heart Failure	68.0	67.0	0.87
Valve Disease	22.3	23.8	0.77
Dilated Cardiomyopathy	13.7	12.7	0.79
Vascular Disease (Aneurysm, Peripheral Occlusive Disease, Vasculitis, Enterectomy)	16.5	16.2	0.95
Carotid Stenosis	10.9	10.2	0.88
Coronary Artery Disease	27.7	27.0	0.90
Previous Myocardial Infarctus	9.1	11.6	0.51
Chronic Kidney Disease	36.1	37.9	0.79
Previous Coronary Intervention	15.3	15.7	0.95
Ischemic Stroke	15.2	15.8	0.91
Intracranial Bleeding	1.3	3.8	0.14
Smoker	19.4	20.6	0.83
Dyslipidemia	50.9	49.2	0.80
Liver Disease	1.6	1.7	0.95
Alcohol Abuse	3.7	4.0	0.90
Thyroid Disease	14.9	13.0	0.65
Poor Nutrition	2.1	3.2	0.59
Previous Cancer	18.0	17.0	0.86
AF	78.7	74.1	0.39
Anemia	20.9	20.0	0.86
≥ NYHA 3	33.0	30.3	0.66
Sleep Apnea Syndrom	25.1	26.3	0.83
Respiratory Failure	8.5	7.5	0.76
COPD	6.2	5.7	0.86
Cognitive Impairment	8.5	8.1	0.92
Pacing Indication			0.36
AV Block Without Sinusal Rythm	43.7	47.1	
AV Node Ablation	32.4	26.5	
Paroxysmal AV Block In Sinusal Rythm With Low Ventricular Stimulation	17.2	13.3	
Sinus Node Dysfunction	3.7	5.7	
AV Block In Sinusal Rythm When AV Synchronisation Is Not Required	3.0	7.5	
LVEF Classification			0.67
1	84.4	82.5	
2	13.0	12.8	
≥ 3	2.7	4.8	
Previous Radiotherapy	2.9	2.7	0.90
Anticoagulation	73.7	69.2	0.45
Corticoid Therapy	3.7	3.1	0.78
Charlson Comorbidity Index	5.0 (1.5)	5.0 (1.8)	0.92
Mean Survival, Years	3.16 (1.4)	3.10 (1.3)	0.67
Length Of Stay (Days)	3.6 (2.2)	4.3 (2.4)	0.007
Duration Of Implantation (Min)	52.3 (14.9)	79.7 (22.2)	<0.001

### Clinical outcomes and events

In unweighted analysis, overall survival was significantly higher in the LP group (HR = 0.58; 95% CI: 0.38–0.87; *p* = 0.008). After weighting, this difference was no longer significant (wHR = 0.73; 95% CI: 0.49–1.07; *p* = 0.11); although Kaplan–Meier curves crossed, the Schoenfeld test was not significant (*p* = 0.072), so the hazard ratio is reported for informational purposes only (Supplementary data Figure 1).

Major clinical events were less frequent in the unweighted LP group (23.1% vs. 34.7%; *p* = 0.038), with no device-related infections reported in the LP group (vs. 2.8% in SLP, *p* = 0.08). After weighting, these differences were attenuated and no longer significant (major clinical events: 25.2% vs. 33.8%, *p* = 0.17; device-related infections: 0% vs. 2.9%, *p* = 0.09). Lead failure occurred in 4.1% of the SLP group. SLP group presented a 4.1% of lead failure and 15.4% and 19.2% of the patients presented at least one cardiac failure event during follow-up in LP and SLP group, respectively (Supplementary data Table 1).

### Costs – implantation and events

Procedure time and length of hospital stay were significantly shorter in the LP group (52.3 ± 14.9 vs. 79.7 ± 22.2 min, *p* < 0.001; 3.6 ± 2.2 vs. 4.3 ± 2.4 days, *p* = 0.007), leading to lower initial hospitalization (€4,264 ± 2,608 vs. €5,142 ± 2,806, *p* = 0.007) and procedural costs (€780 ± 222 vs. €1,189 ± 331, *p* < 0.001). However, device and lead costs were higher with LP (fixed at €6,300 vs. €2,517 ± 446 and €478 ± 123, respectively; *p* < 0.001), resulting in higher total implantation costs (€11,343.9 ± 2,592.8 vs. €9,326.2 ± 2,921.9; *p* < 0.001) (Supplementary data Table 1).

Follow up event costs remained higher in the SLP group over four years, although none of the differences reached statistical significance after weighting (e.g. Year 1: €1,501 ± 4,603 vs. €928 ± 3,882; *p* = 0.27). Four-year total follow-up event costs were also higher with SLP (€2,355 ± 6,244 vs. €1,557 ± 4,447; *p* = 0.16). In terms of total costs, the LP group showed a higher total mean cost although the difference is not significant.

### Cost-effectiveness and sensibility analysis

The mean cost difference between groups was €1,219, for a mean survival difference of 0.06 years (3.16 ± 1.4 vs. 3.10 ± 1.3 years; *p* = 0.64), yielding an ICER of €20,387 per life-year gained.

**Figure 3.**
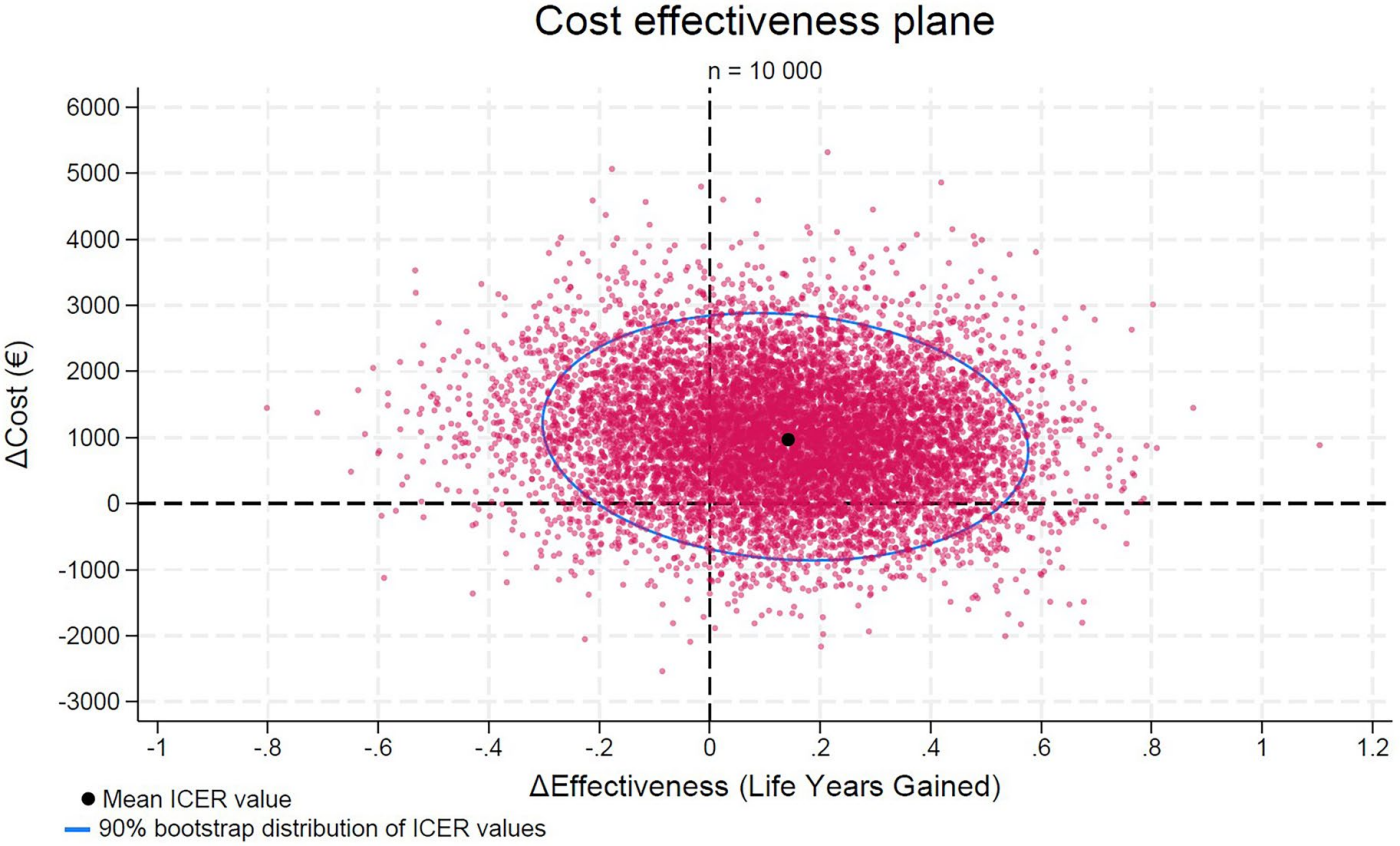
Cost-effectiveness plane.

**Figure 4.**
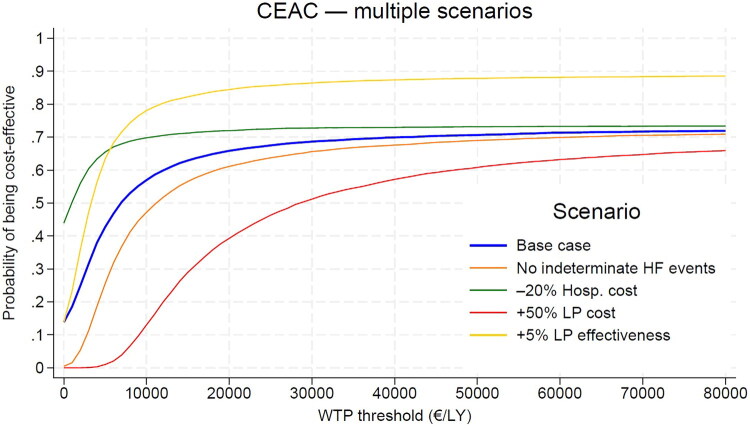
CEAC and one-way sensitivity analysis.

Bootstrapping yielded a mean difference in costs of €1,012 (95% CI: –€743 to €2,951), a mean difference in effectiveness of 0.14 years (95% CI: −0.30 to 0.53), and an ICER of €7,229 (95% CI: –€62,352 to €70,967; Figure 3). The cost-effectiveness acceptability curve indicated a 70% probability of cost-effectiveness at an approximate willingness-to-pay threshold of €35,000 (Figure 4).

Deterministic sensitivity analyses indicated that the ICER was most influenced by variations in LP device cost, survival, hospital stay, and follow-up event costs. Discounting at 4.5% and procedure duration variation had minimal impact. Scenario analyses showed that a 50% increase in Micra VR^®^ cost reduced this probability to about 55%, whereas excluding hospitalizations for heart-failure events of clinically indeterminate origin decrease it to above 65%.In contrast, a 5% gain in LP effectiveness raised the probability to more than 85%, while a 20% reduction in LP hospital stay increased it to around 75%, with dominance (southeast quadrant of the cost-effectiveness plane) observed in 45% of simulations (Figure 4).

## Discussion

In this real-world, retrospective, monocentric analysis using inverse probability weighting based on propensity scores, LP is cost-effective at relatively low threshold values when compared to SLP in a population without formal contraindications to either system. This design allowed a more clinically balanced comparison than most prior studies, which have predominantly focused on selectively implanted, high-risk populations.

Although initial implantation costs were higher for LPs due to device pricing, this was partially offset by significantly lower procedural and hospitalization costs, as well as consistently lower follow-up event-related expenses over a 4-year horizon. After discounting, the ICER was estimated at €20,387 per life-year gained. In the absence of an official WTP threshold in France, commonly accepted European benchmarks (approximately €30,000–€70,000 per QALY or LY) were used for reference [29,30]. Both the base-case and sensitivity analyses indicated that LP Micra VR^®^ was cost-effective, with a probability exceeding 50% at a €35,000 threshold, and with the base-case ICER remaining well within commonly accepted ranges.

The wide dispersion of ICER values observed in bootstrap analyses (95% CI: –€62,352 to €70,967) is largely driven by variability in the mean incremental effectiveness (95% CI: −0.30 to 0.53), including a non-negligible proportion of negative effectiveness values. This effectiveness uncertainty primarily reflects the limited number of deaths in both groups over the 4-year follow-up, as well as the small sample size in the LP arm. Additionally, the crossing of survival curves – likely reflecting higher early procedural risk in the LP group followed by improved long-term outcomes – further contributed to imprecise estimates (Supplementary data Figure 1).

Nevertheless, the likelihood of LP truly resulting in negative incremental effectiveness remains low. Across analyses, LP implantation was consistently associated with a reduction in severe device-related complications, including pocket infections, lead failures, and device-related infections – events known to be associated with substantial morbidity, mortality, and cost. This supports a biologically and clinically plausible long-term benefit. Supporting this, a modest 5% increase in LP effectiveness, corresponding to a bootstrapped incremental survival gain of 0.30 life-years over four years (below estimates reported in another real world study [31]), would raise the probability of cost-effectiveness to 85% at a willingness-to-pay threshold of €20,000 per life-year (Figure 4). This scenario also substantially reduced the proportion of negative bootstrap iterations and aligns more closely with survival trends reported in long-term observational follow-up studies [12,13,32,33].

In contrast, the incremental cost is relatively stable compared with effectiveness, with a mean of €1,219 (95% CI: –€774 to €2,879), and compares favorably with estimates reported in previous modeling studies. The Norwegian Institute of Public Health reported an incremental cost of NOK 16,983 (≈ €1,430) over 10 years in a high-risk population [34], while a national Australian analysis estimated A$4,277 (≈ €3,630) over 17 years based on payer pricing [35]. Variations in reported incremental costs largely reflect differences in device pricing, patient risk profiles, and how long-term complications are accounted for in each model.

Indeed, international variability in device pricing [36] represents the single most important determinant of cost-effectiveness probability. While the estimated LP–SLP device cost differential in our cohort was €3,305, reported differentials across Europe and internationally range from approximately €4,850 to €6,290(31,34,35). In sensitivity analyses, increasing the device cost gap to €6,455 (a 50% increase in LP price) markedly reduced the probability that LP would be cost-effective (Figure 4). Conversely, reductions in hospital length of stay – parameters that consistently favor LP [37–39] – had a substantial economic impact: a 20% reduction in hospitalization duration rendered LP immediately cost-saving in 45% of deterministic simulations.

On the other hand, the relatively low mortality rate observed in our cohort extended the period during which clinically relevant events could occur, thereby increasing cumulative complication-related costs. Although attribution of heart-failure hospitalizations to device type is challenging, complication rates excluding such events remained high compared with published series (LP: 9.6%; SLP: 15.3%). Comparable real-world registries, notably the Micra 5-year post-approval registry, reported lower absolute complication rates for both leadless and transvenous systems, likely reflecting differences in patient selection, procedural expertise and event definitions [40]. Importantly, from a health-economic perspective, absolute event rates are less critical than the difference between device strategies, which is the primary driver of economic outcomes. Consistently, the absolute follow-up event-related cost difference in our study (≈ €800) closely mirrors the difference reported by Makino et al. over a 17-year horizon (≈ €770) [35]. This striking similarity, despite the much shorter follow-up in our study, strongly suggests that complication-related costs in patients eligible for either device type accumulate progressively and may become economically decisive over longer horizons.

This observation is particularly important when interpreted in light of patient selection. Most published Markov-based cost-effectiveness models rely on high-risk cohorts selectively implanted with LPs, reflecting early indications for patients contraindicated for SLPs (e.g. high infection risk or venous occlusion). As highlighted by the Norwegian Institute of Public Health, this selection introduces a structural comorbidity imbalance that limits the generalizability of such models to broader pacing populations [34]. Indeed, in highly comorbid patients, the high risk of death competes with the occurrence of device-related complications, shortening the time window in which complications can both occur and generate costs. Consequently, although LPs reduce complication rates in these patients, their higher upfront cost is less likely to be offset over time, making this population intrinsically less favorable for cost-effectiveness [41].

In contrast, our study focuses on a population eligible for either device type – likely healthier and with better long-term survival prospects. Although not strictly ‘low-risk’, this cohort present a lower *instantaneous* risk of complications compared to high-risk populations [1,42], their cumulative lifetime risk may be comparable – or even higher – due to their extended life expectancy. Consequently, preventing individual complications in this setting is less likely to yield measurable short-term survival benefits, which plausibly explains the modest incremental effectiveness observed over a 4-year horizon. From an economic perspective, however, longer survival increases cumulative exposure to costly device-related events – including generator replacements, lead revisions, and device-related infections – whose incidence remains stable or may even rise over time, particularly in younger and less comorbid patients [6,42–47]. Accordingly, although the observed 4-year event-related cost differential is modest, it becomes a key determinant of cost-effectiveness when analyses are extended beyond short-term follow-up (Figure 4).

This distinction has important implications. In low-risk populations, a long analytic horizon is likely required to demonstrate a clear clinical benefit of LPs in terms of survival or major morbidity. However, clinical benefit may not be the most relevant endpoint in this setting. Instead, economic efficiency may represent the primary driver of value: over a sufficiently long horizon, the cumulative cost of SLP-related complications and reinterventions may exceed the higher upfront cost of LPs, effectively rendering LPs not merely more effective, but potentially less costly overall.

Despite this potential, LPs remain predominantly used in older, highly comorbid patients – precisely those least likely to realize their full long-term economic value. Indeed, and in contrast to our raw data, the majority of comparative studies have reported a paradoxical pattern: LP recipients tend to be younger yet exhibit worse survival in unmatched analyses [21,32,48]. While some guidelines and expert recommendations have begun to support the use of LPs in younger, healthier populations, their adoption in these groups remains limited [49,50].

Taken together, our findings suggest that, in patients at low or intermediate risk, the central question may no longer be whether LPs provide substantial additional clinical benefit over SLPs in the short term, but whether they can deliver superior long-term economic value. In this context, efforts aimed at reducing device costs – through competition, pricing strategies, or technological simplification – may be more impactful than incremental gains in clinical effectiveness. While Micra VR^®^ may not represent the ideal platform for long-term economic optimization due to replacement challenges, newer retrievable and screw-in systems such as Aveir VR^™^ and DR^™^ appear better suited to this paradigm and may facilitate broader, economically sustainable adoption of LP technology in the general pacing population.

## Limitations

Several limitations must be acknowledged when interpreting our findings.

First, owing to the retrospective design, quality-of-life data were not available and QALYs were not estimated. Published real-world evidence suggests LPs provide QoL benefits (e.g. less pain, better function), particularly in younger patients [51], so incorporating utility gains would likely have improved the probability for LP to be cost-effective but would require prospective patient-reported outcomes or valid external utility estimates.

Second, although both LP and SLP implantations were performed at the CHU de Tours, long-term follow-up, especially in the SLP group, was sometimes conducted outside our institution. As a result, some complications may have been incompletely documented or partially captured in the available records. For example, while cases of lead failure were noted in clinical notes, we were not always able to retrieve or code the full diagnostic and cost data. This partial loss of follow-up may have led to an underestimation of complication rates and associated costs in the SLP group.

Third, our cost analysis did not include specific reimbursements for high-cost drugs or medical devices billed outside standard DRG tariffs (so-called ‘*liste en sus*’), because we did not have access to the French National Health Data System (SNDS). Given the higher observed clinical event rate in the SLP group, omission of these additional reimbursements may have led to a slight underestimation of the true cost differential between LP and SLP strategies.

Finally, as a single-center study, our results may not be fully generalizable to other healthcare systems or practice settings. Cost structures, clinical practices, and complication management protocols may vary, highlighting the need for multicenter or international replications to confirm the robustness of our findings. Continued research is needed to validate these findings over extended time horizons and across diverse healthcare settings.

Beyond these limitations, our study also carries broader socio-economic implications. In a context of constrained healthcare budgets, optimizing resource allocation while tailoring treatment strategies to each patient’s individual profile is essential. Just as the concept of personalized medicine is now central for pharmacological innovations, it should also apply to high-value medical devices. Identifying the right device for the right patient is key to maximizing both clinical outcomes and cost-effectiveness. Although modest in scope, our work contributes to this paradigm by providing real-world evidence to inform more personalized and economically sustainable decision-making in device implantation.

## Conclusion

Our single-center, propensity-weighted analysis demonstrates that leadless pacemaker Micra VR^®^ offer a cost-effective alternative to single-lead transvenous systems in patients eligible for either device. Although LPs have higher initial acquisition costs, these are offset by reduced hospital stays, and fewer serious complications, yielding an ICER below accepted European thresholds and an associated 70% probability of LP being cost-effective at a €35,000 per life year threshold. By studying a balanced cohort rather than high-risk patients alone, our findings suggest meaningful long-term benefits with leadless pacing. Future multicenter, prospective trials incorporating quality-of-life measures and extended follow-up are warranted to confirm these findings and guide reimbursement policies toward broader, value-driven adoption of leadless pacing.

## Supplementary Material

Supplementary data 1.docx

graphical abstract.TIF

Supplementary_files new clean.docx

## Data Availability

The datasets generated and/or analyzed during the current study are available from the corresponding authors upon reasonable request.
