# Supplementary material for: Cost-effectiveness of leadless versus transvenous single-chamber ventricular pacing: a propensity-weighted real-world study in France
Source: Ann Med. 2026 Apr 6;58(1):2652657. doi: 10.1080/07853890.2026.2652657 (PMC13055022; doi:10.1080/07853890.2026.2652657)
Supplement: Supplementary data 1.docx [file IANN_A_2652657_SM3408.docx]

Supplementary Data


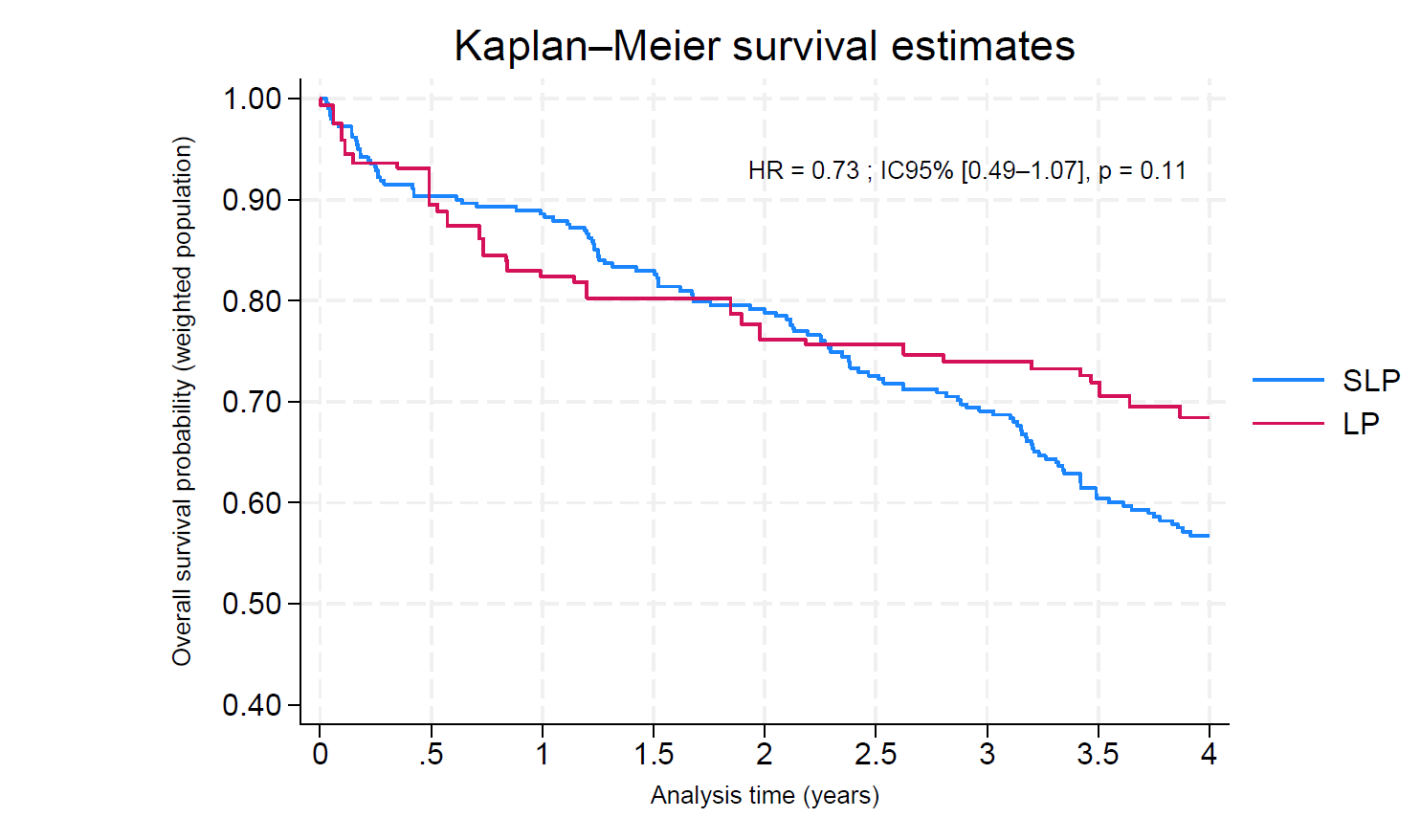


Supplementary figure 1. 4-year survival curve for LP vs SLP after weighting

| Variable | Unweighted | | | Weighted | | |
| --- | --- | --- | --- | --- | --- | --- |
|  | LPM (n = 104) | SCP (n = 248) | *p* | LPM (n = 104) | SCP (n = 248) | *p* |
| **Clinical Events** |  |  |  |  |  |  |
| Device Upgrade | 6 (5.8) | 8 (3.2) | 0.37 | 5.9 | 3.8 | 0.47 |
| Thrombosis | 4 (3.9) | 5 (2.0) | 0.46 | 6.7 | 1.8 | 0.04 |
| Stroke | 3 (2.9) | 10 (4.0) | 0.76 | 2.8 | 3.9 | 0.61 |
| Pulmonary Embolism | 0 (0) | 0 (0) | / | 0 | 0 | / |
| Pneumothorax/Hemothorax | 0 (0) | 3 (1.2) | 0.56 | 0 | 1.2 | 0.27 |
| Arteriovenous Fistula | 0 (0) | 0 (0) | / | 0 | 0 | / |
| Cardiac Failure | 14 (13.5) | 48 (19.4) | 0.19 | 15.4 | 19.2 | 0.49 |
| PM Syndrom | 3 (2.9) | 7 (2.8) | >0.99 | 1.9 | 2.9 | 0.55 |
| Pericardial Perforation | 1 (1.0) | 4 (1.6) | >0.99 | 0.6 | 2.1 | 0.22 |
| Local Infection | 0 (0) | 3 (1.2) | 0.56 | 0 | 1.3 | 0.27 |
| Sepsis | 0 (0) | 4 (1.6) | 0.32 | 0 | 1.8 | 0.2 |
| Endocarditis | 0 (0) | 3 (1.2) | 0.56 | 0 | 1.1 | 0.26 |
| Hematoma | 4 (3.9) | 13 (5.2) | 0.58 | 5.0 | 5.2 | 0.96 |
| Bleeding | 2 (1.9) | 2 (0.8) | 0.59 | 2.8 | 0.8 | 0.18 |
| Lead Failure | 0 (0) | 10 (4.0) | 0.04 | 0 | 4.1 | 0.04 |
| PM Migration | 0 (0) | 0 (0) | / | 0 | 0 | / |
| PM Erosion | 0 (0) | 2 (0.8) | >0.99 | 0 | 0.8 | 0.36 |
| Death | 1 (1) | 3 (1.2) | >0.99 | 0.6 | 1.3 | 0.43 |
| Total Infection | 0 (0) | 7 (2.8) | 0.08 | 0 | 2.9 | 0.09 |
| Total Events | 24 (23.1) | 85 (34.7) | 0.038 | 25.2 | 33.8 | 0.17 |
| **Costs (€)** |  |  |  |  |  |  |
| Cost Of Primo-Hospitalisation Stay | 4485.4 (2990.5) | 5191.9 (2866.7) | <0.001 | 4264.2 (2608.6) | 5142.5 (2806.3) | 0.007 |
| Cost Operating Theatre | 780.8 (226.2) | 1177.6 (304.3) | <0.001 | 779.7 (222.1) | 1189.1 (330.6) | <0.001 |
| Cost Pacemaker | 6300 (0) | 2500.5 (456.1) | <0.001 | 6300 (0) | 2517.0 (446.2) | <0.001 |
| Cost Lead | 0 (0) | 475.2 (118.3) | <0.001 | 0 (0) | 477.7 (122.7) | <0.001 |
| Total Cost Implantation | 11566.3 (2975.7) | 9345.2 (2960.1) | <0.001 | 11343.9 (2592.8) | 9326.2 (2921.9) | <0.001 |
| 1st-Year Event Costs | 574.5 (2789.9) | 1450.3 (4384.6) | 0.02 | 927.8 (3882.2) | 1500.6 (4602.6) | 0.27 |
| 2nd-Year Event Costs | 416.4 (2287.4) | 646.4 (2739.1) | 0.39 | 284.2 (1799.1) | 581.5 (2598.7) | 0.29 |
| 3rd-Year Event Costs | 192.5 (1476.3) | 419.2 (2040.2) | 0.26 | 369.3 (1787.7) | 168.5 (1338.5) | 0.25 |
| 4th-Year Event Costs | 0 (0) | 143.0 (1539.3) | 0.26 | 0 (0) | 136.6 (1475.2) | 0.35 |
| Total Event Costs | 1410.1 (4007.6) | 2432.1 (6409.2) | 0.13 | 1556.6 (4447.3) | 2355.1 (6244.0) | 0.16 |
| Total Cost | 12976.4 (5641.3) | 11777.3 (7420.4) | <0.001 | 12925.2 (5687.4) | 11713.4 (7333.2) | 0.13 |

Supplementary Table 1. Clinical outcomes and associated costs before and after weighting.
