# Supplementary figures and images for: Cost-effectiveness of leadless versus transvenous single-chamber ventricular pacing: a propensity-weighted real-world study in France
Source: Ann Med. 2026 Apr 6;58(1):2652657. doi: 10.1080/07853890.2026.2652657 (PMC13055022; doi:10.1080/07853890.2026.2652657)

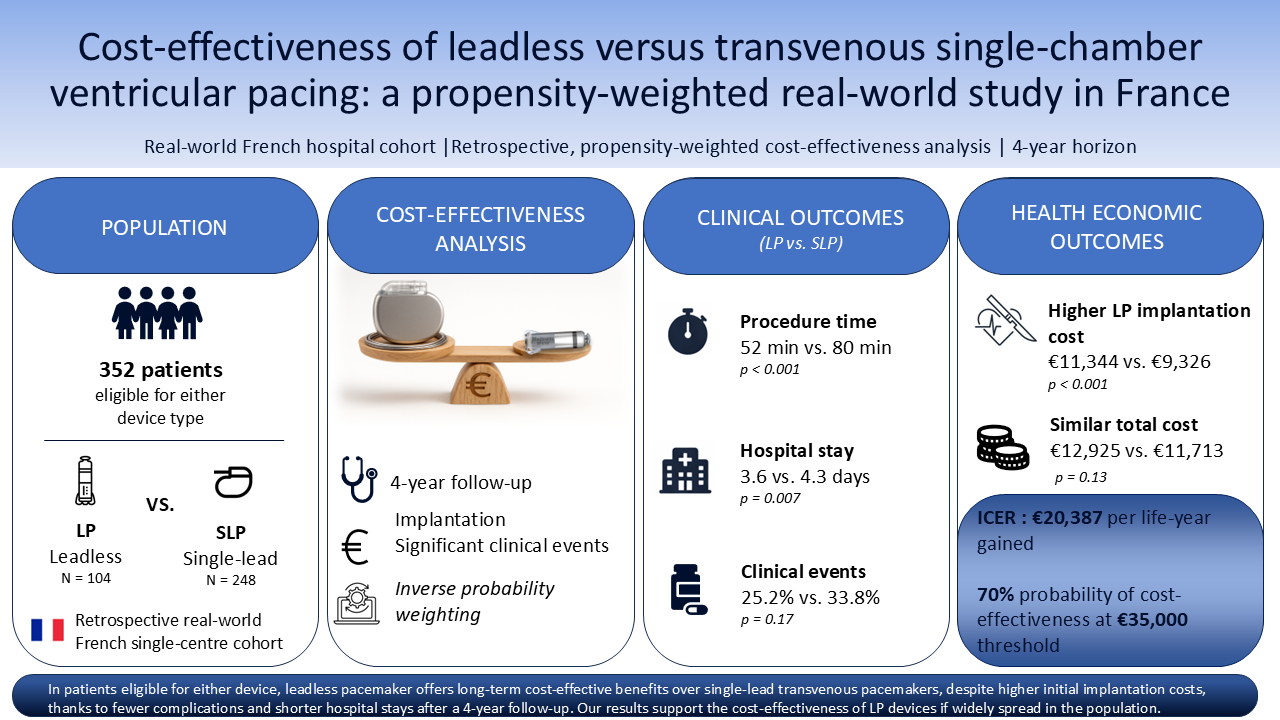

Supplement: graphical abstract.TIF [file IANN_A_2652657_SM3407.tif]
