# Supplementary material for: Cost-effectiveness of leadless versus transvenous single-chamber ventricular pacing: a propensity-weighted real-world study in France
Source: Ann Med. 2026 Apr 6;58(1):2652657. doi: 10.1080/07853890.2026.2652657 (PMC13055022; doi:10.1080/07853890.2026.2652657)
Supplement: Supplementary_files new clean.docx [file IANN_A_2652657_SM3406.docx]

Legends Figures and Tables

Figure 1. Population selection flowchart.

Figure 2. Love plot before and after IPW.

Figure 3. Cost-effectiveness plane.

Figure 4. CEAC and one-way sensitivity analysis.

Table 1. Baseline clinical characteristics before weighting.

Table 2. Baseline clinical characteristics after weighting.
